# Dental Care for a Child with Congenital Hydrocephalus: A Case Report with 12-Month Follow-Up

**DOI:** 10.3390/ijerph18031209

**Published:** 2021-01-29

**Authors:** Yasser Alsayed Tolibah, Chaza Kouchaji, Thuraya Lazkani, Mohammad Tamer Abbara, Saffana Jbara, Ziad D. Baghdadi

**Affiliations:** 1 Department of Pediatric Dentistry, College of Dentistry, Damascus University, Al-Mazzeh St., Damascus P.O. Box 3062, Syria; Firedragoon1994@hotmail.com (Y.A.T.); shazako@yahoo.com (C.K.); 2 Department of Restorative Dentistry and Endodontics, College of Dentistry, Damascus University, Al-Mazzeh St., Damascus P.O. Box 3062, Syria; Dr.thuraya1979@gmail.com (T.L.); tamerabbara@gmail.com (M.T.A.); 3 Department of Preventive Dental Science Division of Pediatric Dentistry, Dr. Gerald Niznick College of Dentistry, University of Manitoba, Winnipeg, MB R3E 0W2, Canada; saffana.jbara@umanitoba.ca

**Keywords:** hydrocephalus, dental care for the chronically ill, facial asymmetry, tooth diseases, oral health, dental care for the disabled, child

## Abstract

Hydrocephalus affects the central nervous system as a result of progressive ventricular dilatation from the accumulation of cerebrospinal fluid in the brain’s lateral ventricles. This paper reports on the oral characteristics of a child with congenital hydrocephalus, discusses her complex dental care needs, and presents dental management of this case. Despite the complex and challenging dental needs, this child received dental treatment in a chairside approach without general anesthesia. A thorough knowledge of the patient’s medical condition, together with expert clinical skills, was indispensable for managing the child and improving the quality and length of her life.

## 1. Introduction

Hydrocephalus (HC) refers to a diverse group of conditions that result from the impaired circulation or absorption of cerebrospinal fluid (CSF). The CSF in the brain’s lateral ventricles causes progressive ventricular dilatation, affecting brain growth and development [1] (HC can happen at any age, but it occurs more frequently among infants, with an incidence ranging from 0.4 to 0.8 cases per 1000 births [2]). The prevalence rate is higher in some developing countries [1]

Hydrocephalus can be congenital or acquired. Congenital hydrocephalus (CHC) may occur due to a ventricular obstruction caused by congenital aqueduct stenosis, neural tube defects (e.g., myelomeningocele and Chiari II malformation), posterior fossa malformation, developmental cysts, and congenital foramen of Monro atresia [1] (Kahle et al., 2016). CHC can be either syndromic or nonsyndromic. The genes implicated in congenital hydrocephalus cause include 2 X-linked (L1CAM and AP1S2) and three autosomal recessive genes (CCDC88C, MPDZ, and EML1) [3]. The maternal environmental risk factors for CHC development include medication and alcohol use during gestation; maternal lifestyle pathologies such as obesity, diabetes, or hypertension; lack of prenatal care; and low socioeconomic status [4]. The causes of acquired pediatric hydrocephalus include inflammation, neoplasm, and vascular disease [1].

The clinical presentation of hydrocephalus varies. Infants may show increased head circumference, bulging of the anterior fontanel, distended scalp veins, broad forehead, deviated eyes downward because of impingement of the dilated suprapineal recess on the brainstem tectum (setting sun sign), and spasticity. In older children, hydrocephalus signs may be subtler, including irritability, lethargy, poor appetite, headache, epilepsy, and vomiting [5].

A gradual change in personality and deterioration in academic productivity suggests a slowly progressive form of hydrocephalus. Children with CH may use difficult words; however, their understanding of abstract concepts is restricted, and their pragmatic conversational skills are weak [6]. Hydrocephalic children are at increased risk for various developmental disabilities. The mean intelligence quotient is reduced, particularly for performance tasks compared with verbal abilities. Many children have abnormal memory and executive function, spatial navigation, learning, and processing speed [1]. Although most hydrocephalic children are pleasant and mild-mannered, some show aggressive behavior. Hydrocephalic children must receive long-term follow-up in a multidisciplinary setting [6].

The diagnosis of HC depends on a combination of characteristic clinical features, MRI imaging of the brain to demonstrate ventriculomegaly (the most crucial diagnostic investigation), and the absence of other causes of encephalopathy [1]. The diagnosis can be made from the dilatation of the lateral brain ventricles during ultrasound examinations after the 20th week of pregnancy. Analyzing CSF biomarkers was suggested as a means to gain insight into the pathophysiology of hydrocephalus and have potential diagnostic and therapeutic value [7].

Despite the possibility of early diagnosis and surgical treatment, the prognosis for CH is still not good, as the perinatal mortality rate is as high as 38% [8]. Most cases of hydrocephalus require extracranial shunts, particularly ventriculoperitoneal shunts (VPSs), which generally consist of Silastic tubing that runs subcutaneously from the head to the abdomen with a valve between the ventricular and distal catheters. The major complications of shunting are occlusion (characterized by headache, papilledema, emesis, mental status changes) and bacterial infection, usually caused by *Staphylococcus epidermidis* and other microorganisms (characterized by fever, headache, and meningismus). The infection is most likely to occur during the first eight weeks after surgery in approximately 10% of cases [9]. Malignant hyperthermia and cerebral venous thrombosis were recently reported after neurologic surgery to insert a VP shunt [10]. Finally, repeated shunt revisions because of shunt failures are associated with a reduction in cognitive functions. Despite reoperation due to high failure rates, shunts have almost the same configuration and design they had when they were introduced in the 1950s [1]. Endoscopic third ventriculostomy (ETV), alone or paired with choroid plexus cauterization (CPC), has been introduced in the U.S. and Canada as an alternative to CSF shunts with favorable results [11].

Dental-related characteristics of patients with HC have been reported in the literature [12,13,14,15]. However, there is little information on the oral health of persons with CHC. Studies cited above have shown that the oral health of children with special-care needs, including those with CHC, is neglected, and this negligence is manifested by the high prevalence of carious teeth, low prevalence of restored teeth, and more biofilm due to poor oral hygiene. Unfortunately, many dental practitioners are not trained to provide dental care for this population, thus causing poor oral health care services. These factors contribute to a low oral-health-related quality of life as reported in the literature concerning children with special needs [16].

Disorders in growth and puberty in children with CHC have been reported to include a craniofacial asymmetry in those patients who always had a shunt on the same side, premature fusion of the cranial sutures (craniosynostosis), and disruptions of dental (teeth) maturation [15,17,18,19]. The facial asymmetry is caused by a moderate growth restriction caused by scar tissue and fibrosis around the shunt on the side where it was inserted. This also adversely affects head posture and muscle balance. Craniosynostosis may result from decompression of the cerebral ventricles using shunts, which interferes with cranial vault growth. The disruption of dental maturation is caused by the alteration of growth hormone and pituitary sex hormones resulting from abnormal intracranial pressure around the hypophysis. A few articles have reported dental care for patients with CHC [14,20,21]. Table 1 summarizes the recommendations for a dental practitioner to follow when managing a child with CHC. The article aims to describe the oral condition of a patient with congenital hydrocephalus (CHC) and present the dental care provided.

## 2. Case Report

**Figure 1:**
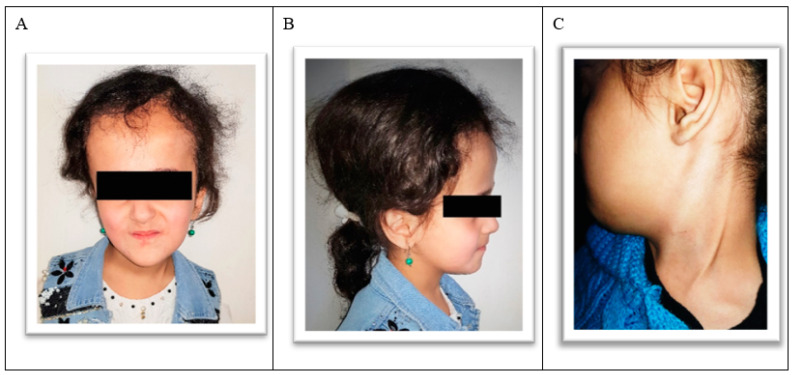
(**A**) Frontal view of the child. (**B**) Lateral view of the child. Note the enlargement in the head’s posterior region due to hydrocephalus. (**C**) The ventriculoperitoneal (VP) shunt in the neck’s left side.

An 8-year-old female with CHC was referred to us for dental treatment due to a lack of cooperation with her general practitioner. During the interview, the mother reported that the child was born with CH. Two months after birth, the child was operated on for a VP shunt placement, which had been replaced twice since then. The spinal column had developed sacralization in the fifth lumbar vertebra and spina bifida occulta in the first sacral vertebra. Currently, the child is taking an anticonvulsive (Tegretol). The mother reported that the child continually suffers from headaches, vomiting, and poor appetite. Despite memory disorders, cognitive problems, and a lack of formal education, the child can eat, change her clothes, and go to the toilet without additional help. An extraoral examination showed an abnormal enlargement of the posterior region of the head. The VP shunt, installed subcutaneously, could be felt on the left side of the head and neck (Figure 1A–C).

**Figure 2:**
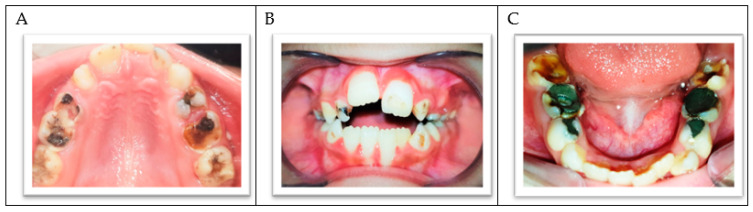
(**A**) The upper arch. (**B**) Frontal view of both arches in occlusion. (**C**) The lower arch.

The intraoral clinical examination showed generalized gingivitis, dental plaque accumulation, and a mild gingival enlargement in the anterior area. Many permanent and primary teeth showed extensive carious lesions. The upper-left central incisor presented signs of hypomineralization and decalcification in the sense of an initial carious lesion (Figure 2A–C). The child was in the transitional dentition with class I molar relationships, showing a mild crowding in lower incisors and a dentoalveolar anterior open bite. The child’s thumb sucking and tongue thrusting habits might have contributed to her malocclusion. The lower primary molars were treated three years ago, but the dental history showed that the child lacked the cooperation ability to complete the treatment correctly (Figure 2C).

**Figure 3:**
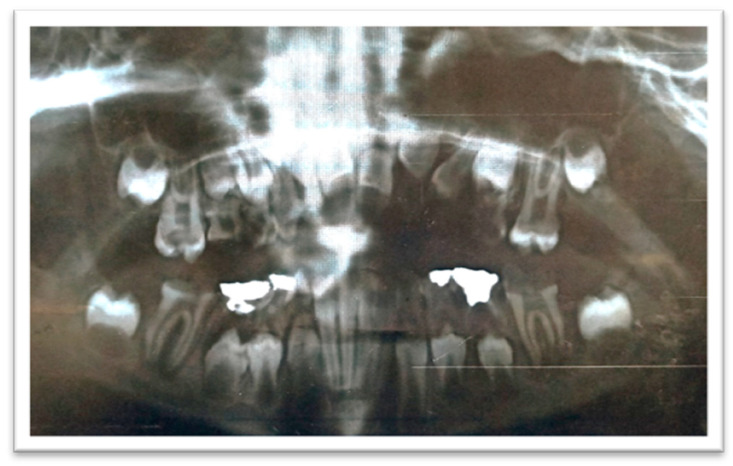
A panoramic radiograph showing teeth #46 and #36 with extremely large carious lesions and periapical radiolucencies, tooth #16 with an extensive carious lesion, and large carious lesions in the upper primary molars.

The radiographic examination showed the absence of the upper-left lateral incisor bud, multiple carious teeth, and apical lesions affecting multiple teeth, including the lower permanent first molars (Figure 3).

Based on medical and dental histories and diagnostic tools, a diagnosis and a problem list were set as shown in Table 2.

A comprehensive treatment plan was established, including

- Toothbrushing and flossing instruction and the establishment of a caries prevention plan: Chlorhexidine mouthwash (0.12%) and gel (0.2%) were prescribed for an initial period of two to three weeks help resolve gingivitis, and the parents were encouraged to supervise the child’s toothbrushing using a high-concentration fluoridated toothpaste.
- Consultation with the child’s physician.
- Treatment of all carious lesions.
- Orthodontic treatment.
- Maintenance and recalls.
- A detailed discussion with patient and parents regarding the treatment plan.

The basic behavioral management techniques were used in all sessions, including tell, show, do (TSD) by explaining with simple terms of what will be done in the upcoming sessions; distraction using mobile games and videos; and positive reinforcement by praising and giving a reward at the end of each session. These techniques enhanced the child’s attitude towards dental treatments as shown by a considerable improvement in behavior. Latex products were avoided because children with neural tube defects are at higher risk for latex allergy [23]. Attention was made to prevent compressing the VP shunt during dental treatment.

## 3. Management

The child’s dental condition was managed over several sessions. The steps presented below outline the order of the treatment procedures applied in the child patient. Each step was completed in one, two, or more sessions as deemed necessary by the treatment team. Flexibility in treatment plans and procedures when treating children with HC is required, and was followed while managing the current child. The treatments were provided after the child became familiar with the clinic’s environment and the dental-care providers.

*Step 1*

The child and parents were instructed on proper tooth brushing with fluoridated toothpaste (high fluoride concentration of 5000 ppm) and flossing to improve the child’s oral health. Full dental records, including radiographs and intraoral photographs, were done. A detailed discussion with the parents regarding the treatment plan was made to explain the difficulties in treating immature and infected permanent teeth as those presented in this case.

*Step 2*

**Figure 4:**
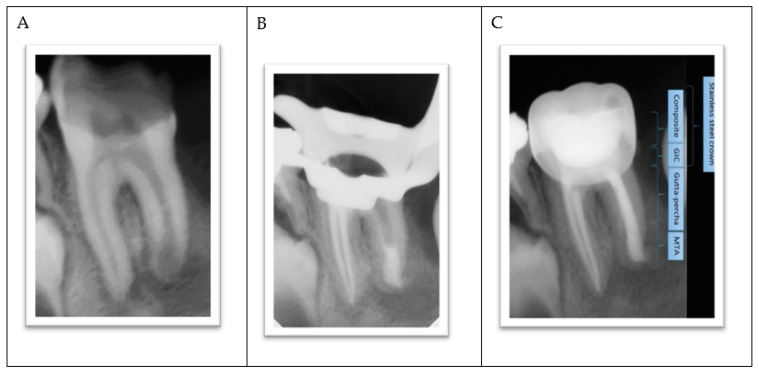
(**A**) Apical lesions with extensive decay in tooth #36. (**B**) Verifying the placement of the MTA plug. (**C**) Completed root canal treatment and placement of a metallic crown.

The lower-left permanent molar (tooth #36) had necrotic pulp and apical lesions. The tooth’s distal root had an open apex, whereas the mesial root showed a closed apex. It was decided to do a traditional root canal in the mesial canals and insert an apical plug using mineral trioxide aggregate (MTA) in the distal root (Figure 4A–C).

- The procedure involved local anesthesia, a rubber dam, and removal of caries.
- Working length was established using a radiograph.
- Copious irrigation with 2.5% sodium hypochlorite NaOCl, followed by minimal mechanical instrumentation of the root canal walls and additional irrigation with Q-mix (Dentsply Sirona) and ultrasonic activation was performed.
- Mesial canals were obturated with gutta-percha using a lateral condensation technique while the distal root was treated with MTA as an apical plug because there was no apical stop.
- A moist cotton pellet was placed in the canal to facilitate MTA setting, and the access cavity was restored using a glass ionomer cement.
- The next day, the setting of the MTA plug was verified, and the remaining portion of the root canal was filled with gutta-percha using lateral condensation. The pulp chamber was cleaned and the coronal access double-sealed with a bonded resin composite.
- A stainless steel crown was inserted.

*Step 3*

**Figure 5:**
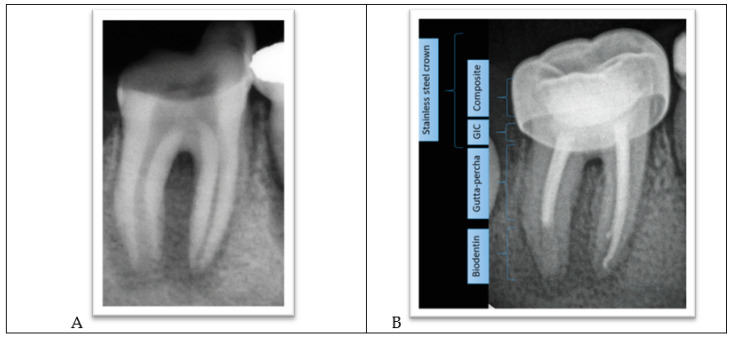
(**A**) Apical lesions with extensive decay in tooth #46. (**B**) Biodentine placement, completed root canal treatment, and placement of a metallic crown.

The lower-right permanent molar (tooth #46) was treated similarly to tooth #36, but Biodentine (Septodont) was used as an apical plug in one session instead of MTA to reduce treatment sessions. The root canal’s remainder was filled with gutta-percha, and the tooth was restored, similar to tooth #36 (Figure 5A,B).

*Step 4*

The upper-right first permanent molar (Tooth #16) had an asymptomatic deep carious lesion.

**Figure 6:**
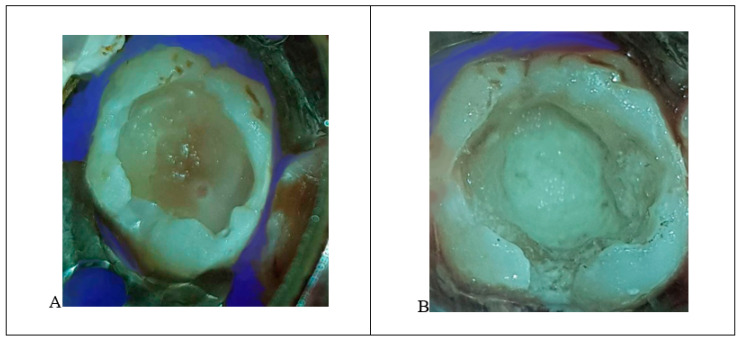
(**A**) A pinpoint pulp exposure in tooth #16. (**B**) MTA placed.

- The procedure involved local anesthesia and rubber dam isolation.
- A pinpoint pulp exposure happened (Figure 6A).
- A coronal pulpotomy was completed by removal of coronal pulp tissue.
- Hemostasis was achieved using 5% sodium hypochlorite and MTA placed as a dressing material over pulp orifices (Figure 6B).
- The tooth was restored like teeth #36 and #46.

*Step 5*

The upper-left first permanent molar (tooth #26) had an asymptomatic superficial carious lesion, which was restored with a metallic crown.

*Session 7*

**Figure 7:**
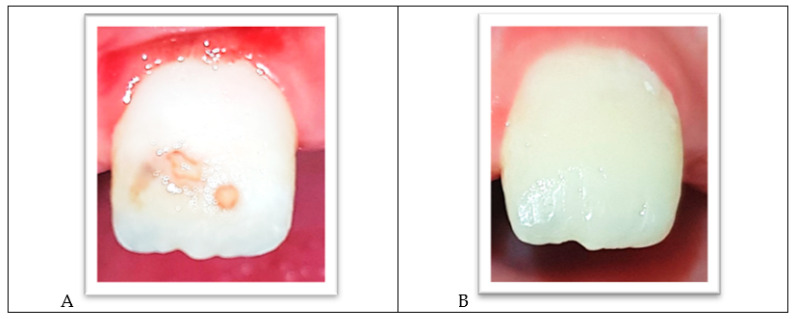
(**A**) Cavitated carious lesion and hypomineralized spots on the buccal surface of tooth #21. (**B**) Resin-bonded restoration after finishing and polishing.

Tooth #21 showed a cavitated carious lesion on the buccal surface paired with hypomineralized spots and was restored with a bonded composite (Figure 7A,B).

*Step 6*

**Figure 8:**
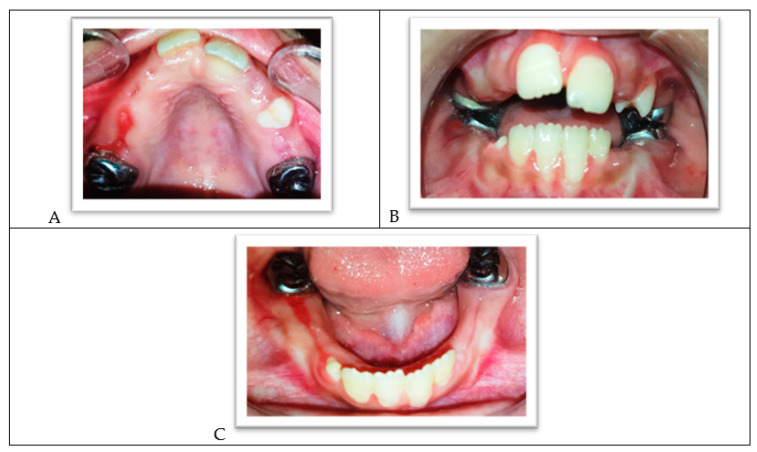
1 month after treatment. (**A**) The upper arch. (**B**) Anterior view in occlusion. (**C**) The lower arch.

A prophylactic antibiotic (50 mg/kg of amoxicillin) was administered before extraction of all primary teeth in a few sessions based on the recommendation of the child’s physician and deemed appropriate by the dentist. Figure 8 shows the dental condition 1 month after the operation.

*Step 7*

After all the dental treatments were performed, interceptive orthodontic treatment was planned to prevent finger sucking and tongue thrusting so that the anterior teeth could re-establish normal eruption and the space of the lower canines and premolars could be preserved. After discussing the advantages and disadvantages of this treatment with the parents, alginate impressions were taken of the child’s upper and lower jaws to make a removable palatal crib for the upper jaw and a fixed lingual arch for the lower.

**Figure 9:**
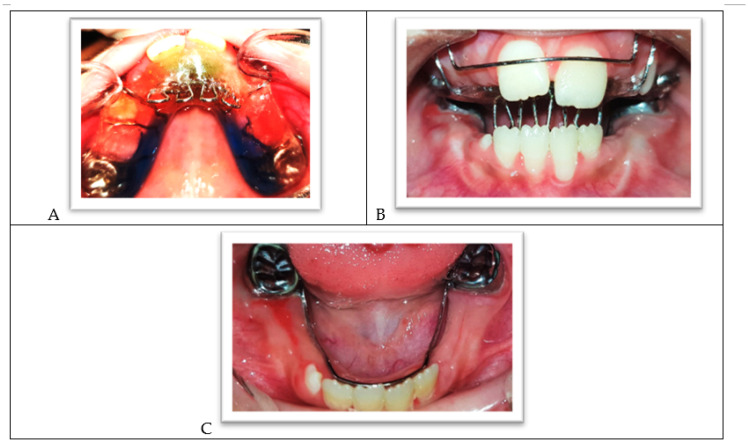
(**A**) The upper arch with a palatal crib. (**B**) The palatal crib in occlusion. (**C**) The lower arch with the lingual holding arch.

In the next session, the palatal crib was placed (Figure 9A,B), and the lingual arch was cemented to the lower permanent molars crowns with a glass ionomer luting cement (Figure 9C). The child and parents were given instructions on cleaning and wearing the palatal crib, and oral hygiene was emphasized. The palatal crib was used for 11 months; it has been slightly modified to accommodate the emergence of the premolars.

### Clinical and Radiographic Follow-Up

**Figure 10:**
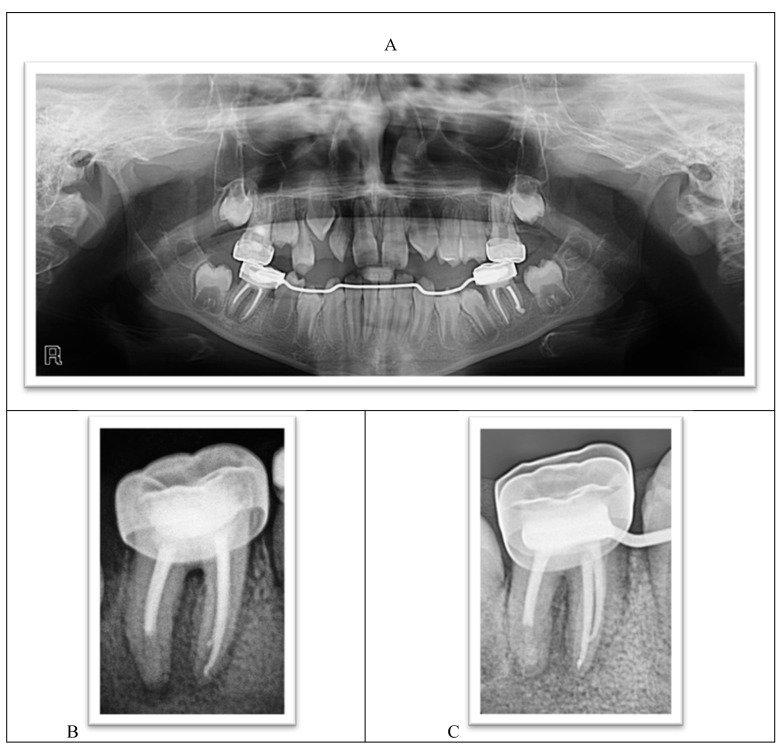

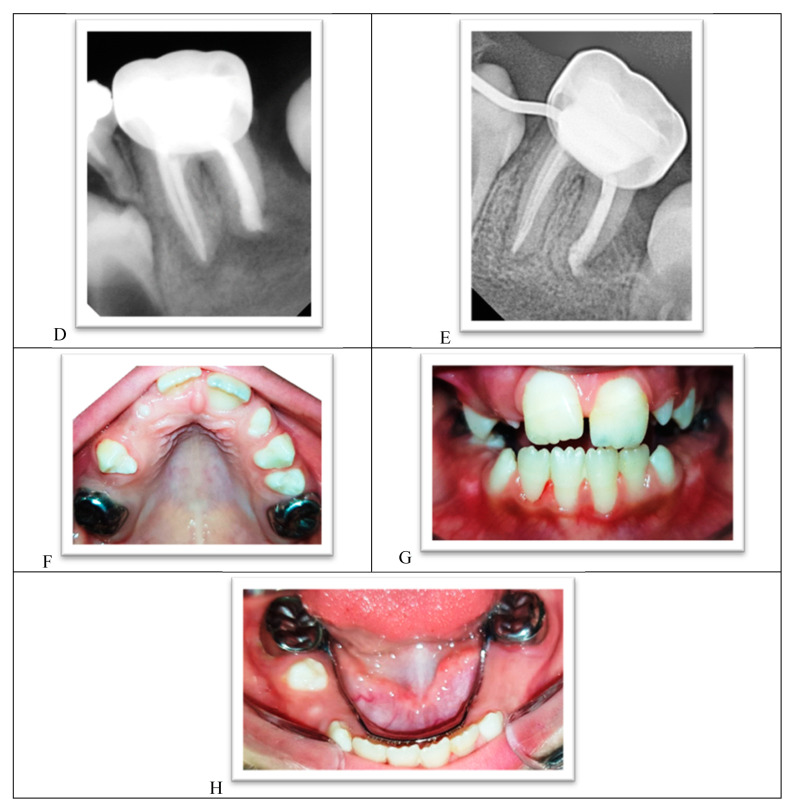
(**A**) A panoramic radiograph one year after treatment. (**B**) The lower-right permanent molar immediately after the treatment. (**C**) The lower-right permanent molar one year after the treatment. Note apical healing. (**D**) The lower-left permanent molar immediately after the treatment. (**E**) The lower-left permanent molar one year after the treatment. Note apical healing. (**F**) The upper arch one year after the treatment. (**G**) Both arches in occlusion. Note the reduction in an open bite. (**H**) The lower arch one year after the treatment.

The parents reported a noticeable improvement in the child’s appetite, the disappearance of dental pain, and improved oral care and tooth brushing habits. Pit and fissure sealants were applied to the premolars as soon as they erupted, a preventive measure recommended for all high caries risk patients. The teeth were asymptomatic and functional 12 months after the operation. The size of the periapical radiolucency diminished, and full healing is expected. There was a calcified bridge under the Biodentine apical plug in the distal root. There was gradual improvement in the anterior vertical dental relationship, improvement in the lower incisor alignment, and increased transverse growth in the lower jaw (Figure 10A–H).

## 4. Prognosis and Discussion

Because the outcomes of the patient’s illnesses are in doubt, the prognosis of caries is guarded due to poor oral hygiene and the child’s special needs but could be improved with better oral hygiene and more frequent recall visits for preventive therapy. The prognosis for improved behavior is also guarded due to the nature of CHC, with lifelong limited intellectual functioning.

The dental treatment of patients with special needs is sometimes performed in a hospital setting under general anesthesia (GA) or sedation. However, it should be noted that individuals with neural tube defects present a high risk of an anaphylactic response during GA [24]. Due to the gradual behavioral improvements after using basic behavioral management methods, the child received dental treatments using local anesthesia with positive results. Treating the child well, praising her positive behavior during treatment sessions, and giving her gifts made the dental visits a favorite thing for her and changed her attitude towards dental treatment.

Comprehensive orthodontic care might also be considered after the eruption of all permanent teeth. As long as the lingual arch appliance is left in place until the second permanent molars fully erupt, the prognosis is good. However, the congenitally missing tooth in this patient’s maxillary arch significantly complicates her care. An alternative treatment plan would have been to wait until permanent dentition eruption to initiate orthodontic treatment; however, regaining the space after the mesial movement of the permanent molars would have made this difficult. The simple use of a lower lingual arch has significant advantages in this type of case. Oral habits such as non-nutritive sucking, bruxing, and abnormal tongue swallowing and positioning can apply forces to the teeth and dentoalveolar structures that may have deleterious effects. The use of anticonvulsant drugs has also been associated with an anterior open bite in children with developmental disabilities, producing respiratory depression, hypoventilation, hypoxia, and obstructive sleep apnea [25,26]). The child must be old enough to understand the need to stop the habit and accept helpful interventions. Treatment is directed toward behavior modification techniques and appliance therapy, such as a palatal crib. Special-needs individuals have high orthodontic needs due to the increased prevalence and severity of malocclusions [27], and the parents of special-needs children are highly motivated to improve their quality of life by improving their appearance and oral function. Unfortunately, special-needs patients are the least likely to receive orthodontic treatment because of major obstacles that may preclude treatment delivery or are encountered during treatment. Different management modalities should be employed to overcome these patients’ behavioral limitations in order to allow orthodontists to gain therapeutic access to them. Patients with special care needs require more chairside time and an increased number of appointments, which means that treating them in a regular orthodontic office among healthy patients is problematic since they would disrupt the schedule. Unlike pediatric dentists, orthodontists are rarely in a position to overcome serious behavioral problems in their patients and, accordingly, find the prospect of treating a special-needs child daunting [28,29]. Cooperation between pediatric dentists and orthodontists and other professionals (e.g., oral surgeons and endodontists) is required to optimize dental care.

There is some controversy concerning antibiotic prophylaxis for patients with shunts. Generally, patients with ventriculoatrial (VA) shunts require antibiotic prophylaxis before dental treatment; those with VP shunts do not [30]. However, some pediatric dentists and neurosurgeons recommend antibiotic prophylaxis for both types of shunts. The reasoning is that the bacteria found in the oral cavity flora are also the bacteria found in shunt infections, including *Staphylococcus* spp. and *Streptococcus* spp. [31]. The research concerning antibiotic prophylaxis is ongoing, and interdisciplinary cooperation between dentists and physicians should be used in treating such patients. Aptekar and Sandor (2006) advise the dental practitioner to err on the side of caution and give antibiotics to patients with shunts [32]. However, reports on dental studies offer contradictory statements on the relationship between hydrocephalic shunt infection and oral maneuvers [13].

Biodentine was used instead of MTA because it has been shown to have better handling properties and, more importantly, a shorter setting time. It also poses a lower risk of tooth discoloration [33]. The shorter setting time of Biodentine helped us with the child’s behavior management by making appointments shorter. QMix is an endodontic irrigating solution aimed at helping eradicate microbes from the root canal system and remove the smear layer. QMix is a 2-in-1 solution containing 2% CHX as a disinfectant and a polyaminocarboxylic acid calcium-chelating agent (17% EDTA) [34]. In the current case, the use of QMix helped reduce treatment time (and, thus, disruptive behavior), as it was claimed to work in 60 to 90 s [24].

### Complications and Alternative Treatment Plans

Complications for this patient could include a relapse of the open bite once the appliances are removed. The relapse could happen because of her poor swallowing pattern or the return of thumb sucking. Although her seizures seem to be well controlled at this point, there is greater concern about how she will tolerate the appliance. If the patient’s behavior had been poor, making it difficult to isolate the tooth restorations or pulp therapy, the alternative treatment would be extraction. Removal of the first permanent molars was an option to avoid the need for space maintenance. After consultation with the dentist, the parents opted to treat the molars rather than have them extracted. Pharmacological management might have required this option for this patient if regular chairside management had not been deemed feasible [35].

## 5. Conclusions

The parents were informed of the importance of achieving optimal dental health in this special-care patient and the recommendations for establishing a dental home to maintain oral health as an essential component of overall general health. As balancing the requirements of a child with special needs can be challenging, the parents were encouraged to work closely with the child’s dentist to put a prevention plan in place so that many potential dental problems can be avoided entirely. Pressing medical issues often take priority, and dental care often takes a back seat. The problem is that children with disabilities or special needs are more likely to develop dental problems compared to otherwise healthy children. Dental care had focused on oral rehabilitation in chairside dental settings, paying close attention to the recommendations related to patients with CHC. Treating patients with CHC requires a thorough background knowledge of the condition paired with expert clinical skills. The overall objective of medical and dental management should be to provide longer life in conjunction with a higher quality of life.

## Figures and Tables

**Table 1:** A summary of recommendations related to the dental management of a child with hydrocephalus [14,21].

Procedure	Recommendation	Modification	Further Information
Medical history	A careful history of general health, associated systemic condition		
Antibiotic prophylaxis	Consider with reservation in invasive dental procedures	May be indicated for ventriculoatrial (VA) shunts rather than ventriculoperitoneal (VP) shunts	Infective endocarditis prophylaxis recommended for patients with underlying cardiac conditions associated with the highest risk of adverse outcome from infective endocarditisfor dental procedures that ✓ involve manipulation of gingival tissue; or ✓ the periapical region of the teeth; or ✓ perforation of the oral mucosa.
VP shunt	Be careful not to compress the catheter during dental treatment	Use pillows and cushions to support the patient’s head	
Intraoral radiographs	Place the radiograph film within a plastic bag 4 cm by 23 cm to avoid the risk of swallowing/suffocation	Let caregiver control the intraoral position of the film	
Prevention	Educate caregivers about the importance of daily oral health care Diet counseling More frequent dental visits More frequent fluoride application or higher fluoride concentration	Demonstrate to caregivers the oral devices used to hold the patient’s mouth open during brushing	

**Table 2:** A diagnosis and a problem list.

Diagnosis	Problem List
• Congenital hydrocephalus	◾ Hyposalivation
• Malocclusion	◾ Poorly compliant parents
• Mild to moderate gingivitis	High caries risk and poor oral hygiene due to [22]:
Extensive carious lesions in teeth #16, #36, #46, #21, and upper primary molars	Special health needs Visible cavitations Enamel demineralization or hypomineralization Lowsocioeconomic status Insufficient oral hygiene measured with the aid of a plaque score High sugar intake measured by a dietary chart Medications that impair saliva flow (Tegretol) Poor toothbrushing performed only on days of dental treatment

## Data Availability

The study did not report any numerical data in the current case report.

## References

[1] Acs G., Cozzi E. (1992). Antibiotic prophylaxis for patients with hydrocephalus shunts: A survey of pediatric dentistry and neurosurgery program directors. Pediatr. Dent..

[2] Akhter R., Hassan N.M.M., Martin E.F., Muhit M., Smithers-Sheedy H., Badawi N., Khandaker G. (2019). Caries experience and oral health-related quality of life (OHRQoL) of children and adolescents with cerebral palsy in a low-resource setting. BMC Oral Health.

[3] Akinwonmi B.A., Kolawole K.A., Folayan M.O., Adesunloye A.M. (2020). Orthodontic treatment need of children and adolescents with special healthcare needs resident in Ile-Ife, Nigeria. Eur. Arch. Paediatr. Dent..

[4] Aptekar A., Sandor G.K. (2006). Point of care. What precautions and measures do I have to consider when treating a patient with ventriculoperitoneal vs ventriculoatrial shunt?. J. Can. Dent. Assoc..

[5] Ausili E., Tabacco F., Focarelli B., Nucera E., Patriarca G., Rendeli C. (2007). Prevalence of latex allergy in spina bifida: Genetic and environmental risk factors. Eur. Rev. Med. Pharmacol. Sci..

[6] Becker A., Shapira J., Chaushu S. (2001). Orthodontic treatment for disabled children--a survey of patient and appliance management. J. Orthod..

[7] Becker A., Shapira J., Chaushu S. (2004). Orthodontic treatment for the special needs child. Semin. Orthod..

[8] Bignardi L., Prates T., De Rossi A., Nelson-Filho P., de Carvalho F.K., de Siqueira Mellara T., de Queiroz A.M. (2018). Strategies and dental care in the treatment of patients with myelomeningocele. Spec. Care Dent..

[9] De Castilho L.S., Abreu M.H.N.G., Pires ESouza L.G.A., Romualdo L.T.A., Souza ESilva M.E., Resende V.L.S. (2018). Factors associated with anterior open bite in children with developmental disabilities. Spec. Care Dent..

[10] De Morais Gallarreta F.W., Bernardotti F.P., de Freitas A.C., de Queiroz A.M., Faria G. (2010). Characteristics of individuals with hydrocephalus and their dental care needs. Spec. Care Dent..

[11] Den Hollander N.S., Vinkesteijn A., Schmitz-van Splunder P., Catsman-Berrevoets C.E., Wladimiroff J.W. (1998). Prenatally diagnosed fetal ventriculomegaly; prognosis and outcome. Prenat. Diagn..

[12] Deshpande A.N., Pradhan N.R., Patel K.S., Mulchandani V.R. (2018). Consequences of Severe Epileptic Attack in a 3-year-old Girl with Congenital Hydrocephalus. Contemp. Clin. Dent..

[13] Garg A., Revankar A.V. (2012). Spina bifida and dental care: Key clinical issues. J. Calif. Dent. Assoc..

[14] Huggare J.A., Kantomaa T.J., Rönning O.V., Serlo W.S. (1986). Craniofacial morphology in shunt-treated hydrocephalic children. Cleft Palate J..

[15] Kahle K.T., Kulkarni A.V., Limbrick D.D., Warf B.C. (2016). Hydrocephalus in children. Lancet.

[16] Kalyvas A.V., Kalamatianos T., Pantazi M., Lianos G.D., Stranjalis G., Alexiou G.A. (2016). Maternal environmental risk factors for congenital hydrocephalus: A systematic review. Neurosurg. Focus.

[17] Kestle J.R., Walker M.L., Strata Investigators (2005). A multicenter prospective cohort study of the Strata valve for the management of hydrocephalus in pediatric patients. J. Neurosurg..

[18] Kinsman S.L., Johnston M.V. (2020). Hydrocephalus. Nelson Textbook of Pediatrics E-Book.

[19] Kuru E., Eden E. (2020). Success of Two Caries Risk Assessment Tools in Children: A Pilot Study With a 3-Year Follow-Up. Int. Q. Community Health Educ..

[20] Limbrick D.D., Baksh B., Morgan C.D., Habiyaremye G., McAllister J.P., Inder T.E., Mercer D., Holtzman D.M., Strahle J., Wallendorf M.J. (2017). Cerebrospinal fluid biomarkers of infantile congenital hydrocephalus. PLoS ONE.

[21] Löppönen T., Saukkonen A.L., Serlo W., Tapanainen P., Ruokonen A., Knip M. (1996). Accelerated pubertal development in patients with shunted hydrocephalus. Arch. Dis. Child..

[22] Moazzam A.A., Nehrer E., Da Silva S.L., Polido J.C., Arakelyan A., Habibian M., Krieger M.D. (2014). The association between dental health and procedures and developing shunt infections in pediatric patients. J. Neurosurg. Pediatr..

[23] Pirttiniemi P., Lahtela P., Huggare J., Serlo W. (1989). Head posture and dentofacial asymmetries in surgically treated muscular torticollis patients. Acta Odontol. Scand..

[24] Pirttiniemi P., Poikela A., Huggare J., Löppönen T. (2004). Dental maturation in children with shunt-treated hydrocephalus. Cleft Palate Craniofac. J..

[25] Poonia A., Chengappa M.D., Mitra R., Jain P., Ghavri T. (2020). Full-mouth Rehabilitation of a Ventriculoperitoneal Shunt-treated Hydrocephalic Pediatric Patient: A Case Report. Int. J. Clin. Pediatr. Dent..

[26] Rajasekharan S., Martens L.C., Cauwels R.G.E.C., Anthonappa R.P. (2018). Biodentine™ material characteristics and clinical applications: A 3 year literature review and update. Eur. Arch. Paediatr. Dent..

[27] Ramasamy C. (2018). Relationship between Dental Procedures and Shunt Infections in Hydrocephalic Patients: A Narrative Review. J. Clin. Pediatr. Dent..

[28] Sacar S., Turgut H., Toprak S., Cirak B., Coskun E., Yilmaz O., Tekin K. (2006). A retrospective study of central nervous system shunt infections diagnosed in a university hospital during a 4-year period. BMC Infect Dis..

[29] Seda G., Tsai S., Lee-Chiong T. (2014). Medication effects on sleep and breathing. Clin. Chest Med..

[30] Schubert-Bast S., Berghaus L., Filmann N., Freiman T., Strzelczyk A., Kieslich M. (2019). Risk and risk factors for epilepsy in shunt-treated children with hydrocephalus. Eur. J. Paediatr. Neurol..

[31] Shaheen R., Sebai M.A., Patel N., Ewida N., Kurdi W., Altweijri I., Sogaty S., Almardawi E., Seidahmed M.Z., Alnemri A. (2017). The genetic landscape of familial congenital hydrocephalus. Ann. Neurol..

[32] Stojicic S., Shen Y., Qian W., Johnson B., Haapasalo M. (2012). Antibacterial and smear layer removal ability of a novel irrigant, QMiX. Int. Endod. J..

[33] Stone S.S., Warf B.C. (2014). Combined endoscopic third ventriculostomy and choroid plexus cauterization as primary treatment for infant hydrocephalus: A prospective North American series. J. Neurosurg. Pediatr..

[34] Tully H.M., Dobyns W.B. (2014). Infantile hydrocephalus: A review of epidemiology, classification and causes. Eur. J. Med. Genet..

[35] Watanabe J., Okamoto K., Ohashi T., Natsumeda M., Hasegawa H., Oishi M., Miyatake S., Matsumoto N., Fujii Y. (2019). Malignant Hyperthermia and Cerebral Venous Sinus Thrombosis After Ventriculoperitoneal Shunt in Infant with Schizencephaly and COL4A1 Mutation. World Neurosurg..

